# Relationship between myocardial performance index and severity of coronary artery disease in patients with non-ST -segment elevation acute coronary syndrome

**DOI:** 10.5830/CVJA-2016-041

**Published:** 2017

**Authors:** Okay Abaci, Cuneyt Kocas, Veysel Oktay, Sukru Arslan, Yusuf Turkmen, Cem Bostan, Ugur Coskun, Ahmet Yildiz, Murat Ersanli

**Affiliations:** Department of Cardiology, Cardiology Institute of Istanbul University, Istanbul, Turkey; Department of Cardiology, Cardiology Institute of Istanbul University, Istanbul, Turkey; Department of Cardiology, Cardiology Institute of Istanbul University, Istanbul, Turkey; Department of Cardiology, Cardiology Institute of Istanbul University, Istanbul, Turkey; Department of Cardiology, Cardiology Institute of Istanbul University, Istanbul, Turkey; Department of Cardiology, Cardiology Institute of Istanbul University, Istanbul, Turkey; Department of Cardiology, Cardiology Institute of Istanbul University, Istanbul, Turkey; Department of Cardiology, Cardiology Institute of Istanbul University, Istanbul, Turkey; Department of Cardiology, Cardiology Institute of Istanbul University, Istanbul, Turkey

**Keywords:** Tei index, Gensini score, acute coronary syndrome

## Abstract

**Objectives:**

We aimed to investigate the relationship between myocardial performance index (MPI) and severity of coronary artery disease, as assessed by the Gensini score (GS), in patients with non-ST-segment elevation myocardial infarction (NSTEMI).

**Methods:**

Ninety patients with an initial diagnosis of NSTEMI were enrolled in our study. They were divided into tertiles according to the GS: low GS <19; mid GS > 19 and ≤ 96; and high GS > 96.

**Results:**

The low-, mid- and high-GS groups included 24, 38 and 28 patients, respectively. Clinical features such as gender distribution; body mass index (BMI); prevalence of diabetes mellitus, hypertension and hyperlipidaemia; and smoking status were similar in the three groups. MPI and isovolumic relaxation time were significantly higher in the high-GS group than in the low- and mid-GS groups (p < 0.001 and p = 0.005, respectively). Furthermore, the high-GS group had a significantly lower ejection fraction and ejection time (p = 0.01 and p < 0.001, respectively). MPI was positively correlated with the GS (r = 0.47, p < 0.001), and multivariate regression analysis showed that MPI was an independent predictor of the GS (β = 0.358, p < 0.001).

**Conclusions:**

Patients with NSTEMI who fall within the high-risk group may be identified by means of a simple MPI measurement.

## Objectives

Non-ST-segment elevation myocardial infarction (NSTEMI) is one of the leading causes of morbidity and mortality, and accounts for high healthcare costs worldwide. The Gensini scoring system, based on angiographic findings, is a valuable method for estimating the severity of coronary artery disease.^1^,^2^ The severity of coronary artery lesions, as assessed by the Gensini score (GS), is associated with long-term mortality and major adverse cardiac event rates.^3^

Doppler-derived myocardial performance index (MPI), also known as the Tei index, is a new diagnostic method and an alternative to ejection fraction (EF) measurements. This index reflects combined systolic and diastolic function and can be defined as the sum of the isovolumic contraction time and isovolumic relaxation time, divided by the ejection time, with a reported normal mean ± standard deviation (SD) value for the left ventricle of 0.39 ± 0.05.^4^ Adverse outcomes are infrequently seen among patients with preserved global ventricular function.^5^

MPI has been identified as a powerful independent predictor of death from all causes in patients with a recent acute myocardial infarction (AMI). In this study, we aimed to determine the association between the severity of coronary atherosclerosis as assessed by the GS and MPI in patients with NSTEMI.

## Methods

The study was a prospective, single-centre analysis of 90 consecutive patients with an initial diagnosis of NSTEMI. Patients who had valvular heart disease, cardiomyopathy, congestive heart failure, previous cardiac surgery, history of percutaneous coronary intervention, chronic kidney disease, hepatic dysfunction, acute respiratory illness, acute infection, chronic inflammatory disease, or complex congenital heart disease were excluded from the study. Patients who were diagnosed with peripheral arterial disease or a coronary artery disease (CAD) equivalent were also excluded.

Data on demographics, established cardiovascular risk factors and medical history were obtained for each patient. Written informed consent was obtained from all subjects, and the investigation conformed to the principles outlined in the Decleration of Helsinki. The local ethics committee approved the study protocol.

## Echocardiographic evaluation

All patients underwent echocardiographic evaluation using a standard protocol on commercially available systems (GE VividiVingmed Ultrasound; Horten, Norway). Comprehensive two-dimensional (2D) and Doppler echocardiographic evaluation were performed with the patient in the left lateral decubitus position before coronary angiography.

Transthoracic echocardiography was performed within the first 24 hours of initial diagnosis. While performing transthoracic echocardiography all patients were monitored by ECG, and echocardiographic parameters were measured with synchronisation by ECG.

In the apical four-chamber view, mitral inflow velocities were measured with the Doppler sample placed at the tip of the valve leaflets, at the left ventricular outflow tract, and below the aortic valve plane. All measurements were averaged over three consecutive cardiac cycles.

Isovolumic relaxation time (IVRT) was measured from closure of the aortic valve to opening of the mitral valve. Isovolumic contraction time (IVCT) was measured from closure of the mitral valve to opening of the aortic valve. Ejection time (ET) was measured from the opening to the closure of the aortic valve on the left ventricular outflow velocity profile. MPI was calculated as the sum of the IVRT and IVCT divided by the ET. Peak velocities of early (E) and late (A) filling were determined according to the mitral inflow velocity curve.

## Angiographic examination

All patients underwent selective coronary angiography via the Judkins technique (IntegrisAllura 9; Philips Medical Systems, Eindhoven, the Netherlands). All angiograms were evaluated by two experienced interventional cardiologists blinded to the clinical baseline characteristics of the patients. In cases of discrepancy, the opinion of a third interventional cardiologist was obtained, and the final decision was made by consensus.

The severity of coronary artery lesions was scored using a modified Gensini scoring system.1 In brief, coronary circulation was divided into eight proximal segments; the percentage by which each lesion in the proximal coronary circulation narrowed the artery was assessed according to the maximal narrowing of the diameter of the artery in all projections.

The extent and severity of proximal coronary disease was assessed by assigning points to each lesion as follows: less than 50% stenosis of the luminal diameter, one point; 50 to 74% stenosis, two points; 75 to 99% stenosis, three points; and total obstruction, four points. The points for each lesion in the proximal coronary circulation were added, and a score for the severity of coronary atherosclerosis was obtained.

According to the modified Gensini scoring system, the degree of coronary stenosis was classified as follows: mild lesions, one to six points; moderate lesions, seven to 13 points; and severe lesions, > 13 points. Patients were divided into tertiles according to the GS: low GS < 19; mid GS > 19 and ≤ 96; and high GS > 96 points.

## Statistical analysis

Statistical analysis was performed using SPSS (Statistical Package for Social Sciences) for Windows version 12 (Chicago, Illinois). Continuous variables are expressed as mean ± SD, and categorical variables are expressed as numbers and percentages. Continuous variables were compared between groups using one-way analysis of variance for normally distributed data, and the chi-squared test was used for nominal variables. Correlations between variables were calculated using the Pearson correlation coefficient. Multiple linear regression analysis was performed to identify the factors related to the GS. A p-value of < 0.05 was considered significant.

## Results

Of the 90 patients included in this study, 24 were assigned to the low-GS group (26.7%), 38 to the mid-GS group (42.2%) and 28 to the high-GS group (31.1%). The demographic and clinical characteristics of the 90 patients with NSTEMI according to the GS are presented in Table 1.

**Table 1:** Baseline characteristics and laboratory findings

**	*Group 1*	*Group 2*	*Group 3*	**
*Variables*	*(n = 24)*	*(n = 38)*	*(n = 28)*	*p-value*
Age (years)	49.4 ± 11.1	54.7 ± 10.3	56.7 ± 9.3	0.035
Male, n (%)	18 (75)	29 (76.3)	26 (92.9)	0.15
Diabetes mellitus, n (%)	6 (25)	13 (34.2)	7 (25)	0.63
Hypertension, n (%)	12 (50)	16 (42.1)	8 (8.6)	0.27
Hyperlipidaemia, n (%)	4 (16.7)	9 (23.7)	2 (7.1)	0.20
Current smokers, n (%)	17 (70.8)	26 (68.4)	21 (75)	0.84
Glucose (mg/dl)	128.6 ± 66.2	136.0 ± 59.7	132.0 ± 54.0	0.91
(mmol/l)	(7.14 ± 3.67)	(7.55 ± 3.31)	(7.33 ± 3.00)	
LDL (mg/dl)	111.1 ± 35.4	131.4 ± 38.8	132.5 ± 35.9	0.07
(mmol/l)	(2.88 ± 0.92)	(3.40 ± 1.00)	(3.43 ± 0.93)	
HDL (mg/dl)	41.1 ± 16.7	36.7 ± 9.0	39.4 ± 8.5	0.34
(mmol/l)	(1.06 ± 0.43)	(0.95 ± 0.23)	(1.02 ± 0.22)	
eGFR (ml/min/1.73 m^2^)	96.8 ± 25.5	96.5 ± 19.5	92.1 ± 21.1	0.66
Haemoglobin (g/dl)	14.2 ± 1.0	13.6 ± 1.7	13.9 ± 1.5	0.31
Leukocytes (× 10^3^/ml)	8513 ± 2506	8826 ± 3527	8813 ± 2288	0.91
Platelet count (× 10^3^/ml)	248 ±68	253 ± 81	233 ± 62	0.55

LDL, low-density lipoprotein cholesterol; HDL, high-density lipoprotein cholesterol; eGFR, estimated glomerular filtration rate.

The mean patient age was significantly higher in the high-GS group than in the low- and mid-GS groups (p = 0.035). However, the groups did not differ significantly with regard to gender; BMI; prevalence of diabetes mellitus, hypertension and hyperlipidaemia; and smoking status.

The echocardiographic parameters of the groups are presented in Table 2. The MPI was higher in the high-GS group of patients than in the low- and mid-GS groups (p < 0.001). IVRT was significantly higher in the high-GS group than in the other groups, and the difference was significant between the high- and low-GS groups, and between the high- and mid-GS groups (p = 0.005). Furthermore, ET was significantly lower in the high-GS group (p < 0.001), whereas the EF was similar in the low- and mid-GS groups, although the high-GS group had a significantly lower EF (p = 0.01).

**Table 2:** Echocardiographic findings in the three groups

**	*Group 1*	*Group 2*	*Group 3*	*p-value*
*Variables*	*(n = 24)*	*(n = 38)*	*(n = 28)*	*p-value*
IVRT (ms)	88.9 ± 18.9	101.7 ± 29.1	113.1 ± 29.9	0.008
IVCT (ms)	67.3 ± 25.1	61.4 ± 31.6	74.0 ± 20.4	0.18
ET (ms)	283.0 ± 24.1	273.9 ± 31.5	241.0 ± 21.4	< 0.001
MPI	0.50 ± 0.11	0.60 ± 0.21	0.72 ± 0.12	< 0.001
E/A	1.0 ± 0.29	0.97 ± 0.46	0.91 ± 0.39	0.31
E/e′	5.8 ± 1.5	6.1 ± 2.1	6.1 ± 1.9	0.87
EF (%)	58.2 ± 3.9	56.4 ± 5.3	53.8 ± 6.2	0.01
Left atrium (cm)	3.30 ± 0.4	3.39 ± 0.4	3.50 ± 0.4	0.25
LVEDD (cm)	4.76 ± 0.3	4.74 ± 0.4	4.84 ± 0.3	0.61
RV (cm)	2.11 ± 0.1	2.21 ± 0.2	2.2 ± 0.1	0.24

IVRT, isovolumic relaxation time; IVCT, isovolumic contraction time; ET, ejection time; MPI, myocardial performance index; E/A, ratio of peak velocities of early (E) and late (A) transmitral filling; E/e′, ratio between early mitral inflow velocity and mitral annular early diastolic velocity; EF, ejection fraction; LVEDD, left ventricular end-diastolic diameter; RV, right ventricle.

**Fig. 1.**
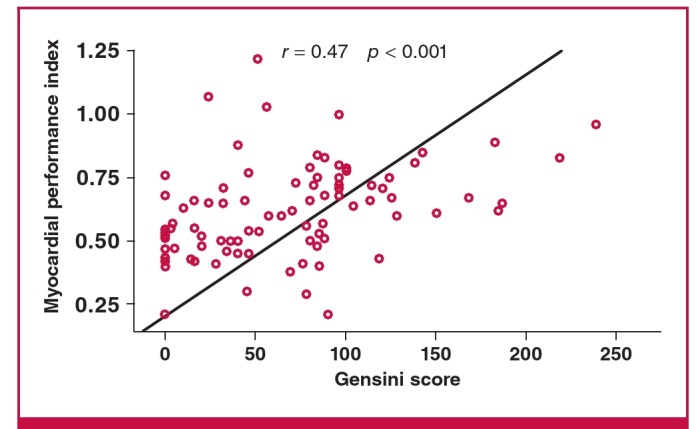
Correlation between MPI and Gensini score.

Correlation analysis was performed to investigate the relationship between the MPI, age and GS. MPI was positively correlated with GS (r = 0.47, p < 0.001; Fig. 1), and age and GS showed a weak positive correlation (r = 0.25, p = 0.01). Multivariate regression analysis for predictors of GS included age and MPI. MPI was identified as an independent predictor of GS (β = 0.358, p < 0.001).

## Discussion

In this study, we found that the risk of significant lesion complexity increased progressively with increasing MPI. According to our results, MPI is an independent predictor of GS, a measure of the severity of coronary artery disease.

Assessment of systolic and diastolic function by non-invasive methods in patients with AMI is of great importance for risk stratification and prognosis.^5^ EF, as determined by routine 2D echocardiography, is the most widely used instrumental parameter for the evaluation of left ventricular function, but this parameter focuses only on systolic function. Both systolic and diastolic functions are frequently affected during an AMI, and therefore, a combined measurement of left ventricular performance may be more useful in assessing overall cardiac function than systolic or diastolic measures alone.

MPI, also known as the Tei index, reflects both systolic and diastolic function of the left ventricle. MPI is calculated using the following formula: (IVCT + IVRT)∕ET.^4^ During the acute phase of an AMI, IVCT and IVRT increase, and when clinical heart failure becomes apparent, the ET decreases. As a result, MPI increases.^6^

MPI is rapidly increased in the early phase of MI and the degree of increment is associated with both mortality and morbidity.^7^ Several studies show that MPI tends to be significantly higher in patients with AMI,^8^,^9^ but these studies do not describe the type of AMI or the severity of coronary involvement.

Sahin et al.^10^ showed that MPI changed in proportion to the severity of CAD in patients with stable CAD, who had an increased prevalence of risk factors such as diabetes and hypertension. However, the increased MPI in that study may have been related to these risk factors, because MPI is reported to be impaired in patients with diabetes and hypertension.^11^

To the best of our knowledge, our study is the first to demonstrate the relationship between MPI and GS in patients with NSTEMI. In this study, the prevalence of risk factors did not differ among groups of patients classified according to the GS, and MPI was an independent predictor of GS.

There are two treatment strategies for patients with NSTEMI: invasive and conservative. Determination of the number of diseased coronary arteries is important in the decision-making process when selecting the course of treatment. The severity of coronary artery disease is associated with mortality in patients with acute coronary syndromes.^12^ In the early period of NSTEMI, measurement of MPI may be useful in the decisionmaking process, for selecting the course of treatment and risk stratification.

Our study has some limitations. First, assessment of coronary angiographic findings was limited to visual interpretation, with inter- and intra-observer variability. Second, the sample size was small and no calculations were made to ensure that the study was adequately powered.

## Conclusion

MPI was an independent predictor of GS in patients with NSTEMI. Patients with NSTEMI who are at high risk may be identified by a simple MPI measurement, which can be useful in the decision-making process for treatment selection and risk stratification.
